# Calixarene-Based Nanostructures for Delivering Coumarin 6 for Tumor-Cell Imaging and Photoinduced Toxicity

**DOI:** 10.1021/acsanm.5c05263

**Published:** 2026-02-06

**Authors:** Loredana Ferreri, Giuseppe Granata, Giuseppe Forte, Melchiorre Cervello, Antonella Cusimano, Salvatore Petralia, Grazia Maria Letizia Consoli

**Affiliations:** † 9327 Institute of Biomolecular Chemistry - C.N.R, Catania 95126, Italy; ‡ Department of Drug and Health Sciences, 9298 University of Catania, Catania 95125, Italy; § Institute for Research and Biomedical Innovation - C.N.R, Palermo 90146, Italy

**Keywords:** supramolecular chemistry, fluorescence imaging, cancer cell, nanotheranostics, calixarene, coumarin 6, drug delivery, photodynamic therapy

## Abstract

Fluorescence imaging techniques are emerging as safer and more sensitive alternatives to radionuclide-based approaches for cancer diagnosis. Entrapping fluorophores that also act as photosensitizers within nanocarriers is an effective strategy to enhance their stability, performance, and selective delivery to tumor tissues, enabling the development of advanced nanosystems for cancer cell imaging and theranostic applications. In this work, we report a fluorescent nanosystem obtained by entrapping Coumarin 6 in biocompatible nanostructures formed through the self-assembly of an amphiphilic calix[4]­arene derivative functionalized with choline ligands for targeting tumor cells. The nanosystem was characterized using diverse techniques, which confirmed nanoscale organization, colloidal stability, excellent resistance to freeze-drying, good fluorescence quantum yield, and visible-light-triggered photodynamic activity, as evidenced by methylene blue photodegradation. Molecular modeling simulations provided mechanistic insight into the host–guest interactions governing Coumarin 6 stabilization in the nanocarrier. The nanosystem selectively imaged breast carcinoma (MCF-7 and MDA-MB-231) and hepatocarcinoma (Hep3B and SNU398) cells that overexpress the choline transporter, while negligible uptake was observed in nonmalignant human fibroblasts (HuDe cells). Intracellular fluorescence intensity correlated with choline transporter expression levels, and the competitive inhibition assay supported a transporter-mediated cellular uptake mechanism. Upon biocompatible visible-light irradiation, the nanosystem effectively induced cancer cell death through activation of the Coumarin 6 photosensitizer. The integration of targeted cancer cell imaging and phototriggered cytotoxicity highlights this fluorescent nanosystem as a promising photoresponsive nanotheranostic candidate.

## Graphical abstract

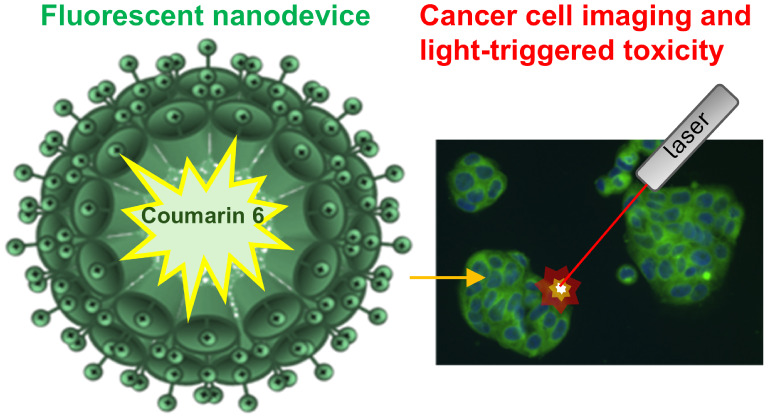

## 1 Introduction

Fluorescence imaging is an emerging technique for early cancer diagnosis and precision therapy.^1,2^ It is expected to play an increasingly important role in the clinical management of cancer patients and in cost-effective, image-guided personalized medicine. Compared with currently used imaging techniques, such as radiography, computed tomography (CT), magnetic resonance imaging (MRI), and positron emission tomography (PET), fluorescence imaging offers the advantages of noninvasiveness, high spatiotemporal resolution, high sensitivity, and real-time monitoring, as it typically does not require image reconstruction and extensive postprocessing.^3^ Moreover, unlike the aforementioned techniques, fluorescence imaging has proven to be a powerful intraoperative tool for guiding precision surgery. A fluorescent probe into the tumor site enables real-time visualization of tumor cells, allowing for more complete tumor resection.^4^ Currently available commercial fluorescent probes rely on specific monoclonal antibodies labeled with fluorescent dyes, which provide high sensitivity and selectivity but are very expensive. Therefore, there is growing interest in the development of novel fluorophores and enabling technologies.

Nanotechnology is poised to revolutionize fluorescence-based diagnostic imaging. Nanocarriers can overcome many limitations of conventional optical imaging methods.^5,6^ They can solubilize hydrophobic dye molecules in aqueous media, improve their bioavailability, enhance stability and circulation time, and enable targeted delivery. Since many fluorophores also function as photosensitizers, their vehiculation in nanocarriers represents a highly promising strategy for developing advanced nanotheranostic agents for simultaneous diagnosis and photodynamic therapy (PDT).^7^ The light-triggered therapeutic action, coupled with intrinsic fluorescence for imaging, enables precise spatiotemporal control over treatment, while simultaneously providing real-time diagnostic monitoring. Fluorophores entrapped in nanocarriers, owing to nanoscale size and high surface-to-volume ratio, can preferentially accumulate in tumor tissues through the enhanced permeability retention (EPR) effect^8^ and interact with target cells via a large surface contact area. Furthermore, the presentation of multiple ligand units on the nanocarrier surface can enable more effective and selective binding to tumor cell receptors through a multivalency effect.^9^ Receptor-mediated cellular uptake ensures targeted delivery of bioactive molecules to viable cells, minimizing off-target effects and enhancing both safety and efficacy.^10^

Coumarin 6 (C6) is a coumarin derivative featuring a benzothiazolyl group at position 3, and belongs to the 7-diethylaminocoumarin series. C6 is an amphipathic dye widely used in biological applications due to its high biocompatibility, green fluorescence emission, photochemical stability, and lipid-like structure. These properties make C6 suitable for specific staining of eukaryotic cell components, detection of biologically relevant analytes, and *in vivo* tracking and transport mechanism studies. For instance, C6 has been employed to demonstrate the effective skin penetrability of PLGA-based nanoparticles and the efficacy of liposome in drug delivery to the retina.^11^ Entrapment of C6 within delivery systems circumvents limitations associated with its poor water solubility, providing a fast, reliable, and high-quality tool for cell imaging. Several studies have reported greater efficiency of C6 when entrapped in nanocarriers compared to its free form.^12^ Polymeric nanoparticles composed of pluronic F127 and vitamin E-TPGS encapsulating C6,^13^ as well as inclusion complexes of C6 with β-cyclodextrin,^14^ have been successfully tested for rapid optical imaging of various tumor cell types. Nanoformulated C6 has been proposed for fluorescence tracking in different brain regions^15^ and in colonoscopy applications,^16^ via intranasal and intrarectal administration, respectively. Coumarins can also act as photosensitizers for photodynamic therapy (PDT)^17^ but suffer from poor aqueous solubility, aggregation, low photostability, and rapid clearance. Entrapment of hydrophobic photosensitizers in nanoscale delivery systems is a strategy to enhance their therapeutic potential.^18^

Calix­[*n*]­arenes are a class of polyphenolic macrocyclic compounds widely studied in supramolecular chemistry.^19^ They feature a hydrophobic cavity capable of complexing a variety of guest molecules and exhibit remarkable synthetic versatility, enabling applications across materials and life sciences.^20^ Water-soluble calixarene derivatives have been explored as agents in drug discovery^21^ and drug delivery.^22,23^ Calixarene derivatives can self-assemble into nanoscale micelles,^24^ vesicles,^25^ and solid lipidic nanoparticles,^26^ functioning as nanocarriers with multiple binding sites, including the calixarene cavity, palisade layer, hydrophobic micellar core, aqueous vesicular compartment, and surface functional groups. Previously, we developed an amphiphilic calix[4]­arene derivative (Chol-Calix) bearing choline ligands at the upper rim and long C12 alkyl chains at the lower rim.^27^ Chol-Calix, spontaneously self-assembles into micellar nanostructures in a biomimetic medium (10 mM PBS, pH 7.4). These nanostructures can entrap various hydrophobic drugs, including curcumin,^28^ silybin,^29^ nitric oxide donor,^27^ photosensitizers,^30^ and antibiotics,^31^ and their efficacy as a drug delivery system has also been validated in animal models of uveitis,^28^ age-related degenerative maculopathy (ADM),^29^ and psoriasis.^32^ Rodik et al. also demonstrated the potential of Chol-Calix as a nanocarrier for gene delivery.^33^

Choline transporters play a role in choline metabolism, which is often altered in cancers such as breast, prostate, ovarian, pancreatic, and hepatocellular carcinoma.^34–37^ To meet the increased choline demand, tumor cells typically upregulate choline transporter genes, resulting in faster choline uptake compared to normal cells.^38^ This feature has been exploited in the development of radiolabeled choline analogs for PET imaging of cancer.^39,40^

In this study, we investigated the ability of the Chol-Calix nanostructure to entrap C6 and evaluated the potential of the resulting fluorescent nanosystem as an agent for tumor cell imaging and nanotheranostics. The nanosystem was thoroughly characterized for its physicochemical and photophysical properties, and the interactions of C6 with the calixarene-based nanocarrier were investigated by molecular modeling simulations. Choline-mediated targeted tumor cell imaging was assessed in breast carcinoma (MCF-7 and MDA-MB-231) and hepatocarcinoma (Hep3B and SNU398) cell lines, which express different levels of choline transporters, and compared to nonmalignant human dermal fibroblasts (HuDe cells). Furthermore, the photoinduced cytotoxic effect was evaluated in breast carcinoma cells.

## 2 Experimental Section

### 2.1 Materials

Reagents were purchased from Sigma-Aldrich and used without further purification.

### 2.2 Synthesis and Characterization of Chol-Calix

Chol-Calix was synthesized as reported in the literature,^27,28,33^ and as detailed in the Supporting Information. The structure of Chol-Calix was confirmed by ^1^H NMR spectroscopy (Bruker Avance, 400.13 MHz), showing characteristic signals consistent with the expected and previously reported data.^27,33^

### 2.3 Preparation of the Fluorescent Chol-Calix/Coumarin 6 Nanosystem

Coumarin 6 (2.5 mg) was added to a colloidal solution of micellar Chol-Calix in a PBS medium (10 mg/10 mL, 0.6 mM). The mixture was sonicated for 15 min, stirred at room temperature in the dark for 3 days, and then centrifuged at 10,000 rpm for 15 min. The supernatant was passed through a 0.2 μm GHP filter to give a yellow colloidal dispersion.

### 2.4 Characterization of the Chol-Calix/C6 Nanosystem

UV–vis spectra were recorded on an Agilent Technologies 8453 UV–vis spectrophotometer. The amount of loaded C6 was determined from the absorbance at 470 nm of the sample diluted in PBS:MeOH (1:2, *v/v*), by referring to a calibration curve in the same solvent mixture. The calibration curve was obtained by dissolving 0.153 mg of C6 in MeOH (1 mL) and adding 10 μL aliquots to a mixture of PBS (400 μL) and MeOH (800 μL). *R* ^2^ = 1, molar coefficient extinction equal to 38,055 M^–1^cm^–1^.

Drug loading capacity (%) was calculated using the following formula:

1 $$LC\;\left(\%\right)=\frac{mass\;of\;entrapped\;C6}{mass\;of\;entrapped\;C6+mass\;of\;nanocarrier}\times 100$$

Fluorescence emission spectra were recorded on a Horiba-Jobin-Yvon fluorescence spectrometer, λ_exc_ = 470 nm, λ_em_ = 507 nm. The quantum yield of C6 was determined by using fluorescein as the standard (Φ = 0.91) according the following equation:

2 $$\Phi_{\mathrm{un}}=\Phi_{\mathrm{std}}\left[I_{\mathrm{un}}/I_{\mathrm{std}}\right]\left[{\eta_{\mathrm{un}}}^{2}/{\eta_{\mathrm{std}}}^{2}\right]$$

where Φ_un_ = quantum yield of the unknown dye, Φ_std_ = quantum yield of the standard dye, *I*_un_ = fluorescence intensity of the unknown dye, *I*_std_ = fluorescence intensity of the standard dye, η_un_ = refractive index of the unknown dye solvent, and η_std_ = refractive index of the standard dye solvent.

### 2.5 Dimensional and Z Potential Analyses

The measurements were carried out through dynamic light scattering and electrophoretic light scattering by using a ZetaSizer NanoZS90 (Malvern Instruments, Malvern, UK), equipped with a 633 nm laser, at a scattering angle of 90° and at 25 °C temperature. The size of the particles was calculated from the diffusion coefficient by using the Stokes–Einstein equation:

3 $$D=\frac{k_{B}T}{6\pi \eta R_{H}}$$

where *D* is the diffusion coefficient, *k* is the Boltzmann constant, *T* is the absolute temperature, η is the solvent viscosity, and *R*_*H*_ is the hydrodynamic radius.

The zeta potential (ζ) was calculated by using Henry’s equation:

4 $$U_{E}=\frac{2\varepsilon \zeta }{3\eta }f\left(Ka\right)$$

where *U*_*E*_ is the electrophoretic mobility, *ε* is the dielectric constant, *f*(*Ka*) is Henry’s function, and η is the viscosity.

### 2.6 TEM Analysis

The morphology of the Chol-Calix nanostructures was analyzed under a transmission electron microscope (TEM, JEOL, Japan) using an accelerating voltage of 200 kV, at room temperature. The unstained specimens were prepared by placing a drop of Chol-Calix colloidal solution on copper TEM grids coated with a thin, amorphous carbon film. The grids were dried in air, and the dried specimens were examined.

### 2.7 Molecular Modeling

The simulation study was performed using four different Chol-Calix/C6 structures with different C6 orientations, as described in the Supporting Information. The Chol-Calix/C6 geometries were optimized at the CAM-B3LYP/6-31G­(d) level, incorporating the Polarizable Continuum Model (PCM) to account for implicit solvent effects, which is water. Noncovalent interactions in molecular assemblies include dispersion forces, which arise from instantaneous charge fluctuations, as well as dipole-induced dipole and dipole–dipole interactions, including hydrogen bonding. The CAM-B3LYP hybrid exchange-correlation functional is particularly effective in accurately predicting the strength of these noncovalent interactions.^41–47^ The optical UV–vis spectra were computed for the optimized structures using the time-dependent DFT (TD-DFT) approach,^45,46^ employing the 6-31G­(d) basis set and the CAM-B3LYP functional. Finally, Gibbs free energies in solution were calculated at the same theoretical level using the SMD solvation model.^47^

### 2.8 Photosensitizing Experiments

An aliquot of Chol-Calix/C6 (2 mL, Abs_470 nm_ = 0.7) was mixed with a methylene blue solution (Abs_665 nm_= 0.51) and irradiated under stirring with a CW laser source (470 nm, 800 mW). The experiments were replicated three times in aerated and deaerated conditions (15 min with argon gas). The optical absorption spectra were recorded at different irradiation times (from 0 to 60 min).

### 2.9 Cell Cultures

Primary dermal human cell line HuDe (BS PRC 41) was purchased from the Istituto Zooprofilattico Sperimentale of Lombardia and Emilia Romagna (Brescia, Italy) and maintained in culture with DMEM medium (Gibco, Life Technologies, Monza MB, Italy), supplemented with heat-inactivated 10% Fetal Bovine Serum (FBS, Gibco, Life Technologies) and 1% antibiotics (penicillin 100 U/mL, and streptomycin sulfate 100 mg/mL, from Invitrogen, Carlsbad, CA, USA). The human breast adenocarcinoma cell lines MCF-7 and MDA-MB-231 were obtained from ATCC (Rockville, MD, USA) (HTB-22 and HTB-26, respectively) and cultured in Dulbecco’s Modified Eagle Medium (DMEM) supplemented with 10% heat-inactivated FBS, 2 mM l-glutamine, 100 U/mL penicillin, and 100 μg/mL streptomycin. The human HCC cell lines HepG2, Hep3B, SNU398, and SNU475 were acquired from the American Type Culture Collection (ATCC) (HB-8065, HB-8064, CRL-2233, and CRL-2236, respectively), Huh7 cells were a gift from Prof. M. Levrero (Sapienza University of Rome, Rome, Italy), and PLC/PRF/5 cells used in this study were a gift from Prof. O. Bussolati (Unit of General and Clinical Pathology, Department of Experimental Medicine, University of Parma, Parma, Italy) and were maintained as previously described.^48,49^ The cell lines were authenticated by using short tandem repeat profiling (BMR Genomics, Padua, Italy).

### 2.10 Western Blotting for Choline Receptor Detection

35 × 10^4^ cells per well were plated in 6-well plates. After 24 h whole cellular lysates from cells were obtained using RIPA buffer (Cell Signaling Technologies Inc., Beverly, MA, USA) and Western blot analysis was performed using the methodology for the Odyssey infrared imaging system (LI-COR Biosciences, Lincoln, NE, USA), as previously described.^50^ Membranes were scanned and analyzed with an Odyssey infrared imaging system (LI-COR Biosciences) using Odyssey 3.0 imaging software. Primary antibodies: β-actin (Sigma-Aldrich, Milan, Italy) and SLC44A1/CD92 (Bioss Antibodies, Woburn, MA, USA).

### 2.11 Cellular Uptake

Ten × 10^3^ HuDe cells, 20 × 10^3^ MCF-7 cells, 10 × 10^3^ MDA cells, 20 × 10^3^ Hep3B cells, and 30 × 10^3^ SNU398 cells were plated in complete medium on 8-well chamber slides. After 24 h, cells were incubated with Chol-Calix/C6 (25 μM Chol-Calix, 0.77 μM C6) for 1 h at 37 °C, under a controlled, humidified atmosphere containing 5% CO_2_, and at 4 °C on ice. Thereafter, cells were washed two times with PBS, containing calcium and magnesium ions, fixed in 4% of paraformaldehyde (PFA) for 10–15 min at room temperature, and then cells were analyzed for fluorescence. As negative controls, cells were treated with vehicle alone. Fluorescence images were acquired using an Olympus inverted microscope at the GFP channel (λ_em_ 508 nm, λ_exc_ 470 nm) for Chol-Calix/C6, and the DAPI channel (λ_em_ 455 nm, λ_exc_ 345 nm) for nuclei.

### 2.12 Cellular Uptake of Chol-Calix/C6 in the Presence of Choline Excess

MCF-7 cells were incubated for 30 min with the different doses of choline (40, 80, 100, and 200 mM), then cotreated with Chol-Calix/C6 (25 μM Chol-Calix, 0.77 μM C6) for 1 h. The excess of Chol-Calix/C6 was washed out three times with PBS. Then cells were fixed with 4% PFA and fluorescence was analyzed by fluorescence microscopy. The quantification of intracellular fluorescence levels due to Chol-Calix/C6 was performed by ImageJ software. Fluorescence intensity was normalized to cell number given by the DAPI staining of the nuclei. Data shown are means ± S.E.M., representative of three independent experiments, and they are expressed as percentage of fluorescence in MCF-7 cells incubated with Chol-Calix/C6 alone. *p < 0.05, compared to control MCF-7 cells not incubated with free choline. Results were considered significant with *p* < 0.05 (*t* student analysis).

### 2.13 Irradiation of Chol-Calix/C6-Loaded Breast Cancer Cells

MCF-7 cells (5 × 10^3^/well) were plated, in complete culture medium without phenol red, on a 96-well plate. Cells were plated in a scheme that minimizes any interference between the different treatments. In more detail, cells were plated in triplicates for each experimental point, and moreover each group of three wells was spaced from each other by at least two wells. After 24 h, cells were incubated with Calix-Chol/C6 (25 μM Chol-Calix, 0.77 μM C6) for 1 h at 37 °C, under a controlled humidified atmosphere containing 5% CO_2_. Then cells were irradiated with a laser light (λ 470 nm) for 10 min in a heating plate at 37 °C. Thereafter, cells were placed in an incubator under a controlled humidified atmosphere containing 5% CO_2_. After 24 h, cell viability was assessed by MTS assay, using the CellTiter Aqueous One Solution kit (Promega, Madison, WI, USA) according to the manufacturer’s instructions.

## 3 Results and Discussion

### 3.1 Entrapment of C6 in the Chol-Calix Nanocarrier and Characterization of the Chol-Calix/C6 Nanosystem

The calix­[4]­arene derivative Chol-Calix (Figure 1A) was synthesized as previously reported,^27,28,33^ detailed in the Supporting Information and depicted in Figure S1. Briefly, the phenolic OH groups of commercially available *p-*H-calix­[4]­arene were functionalized with C12 alkyl chains. *p*-Formyl groups were introduced at the calix[4]­arene upper rim and reduced to *p*-hydroxymethyl groups that were converted to *p*-chloromethyl groups. Subsequent reaction with dimethylaminoethanol afforded the final amphiphilic, polycationic choline-calix[4]­arene derivative (Chol-Calix, Figure 1A). Its structure was confirmed by ^1^H NMR spectrum (Figure S2), which displayed signals consistent with complete functionalization of the macrocyclic scaffold, bearing four dodecyl aliphatic chains at the lower rim (phenolic OH groups) and four choline-like substituent groups at the upper rim (aromatic rings) of the macrocycle. The presence of an AX system for the bridged methylene groups indicated a calix[4]­arene framework locked in the cone conformation.

**Figure 1:**
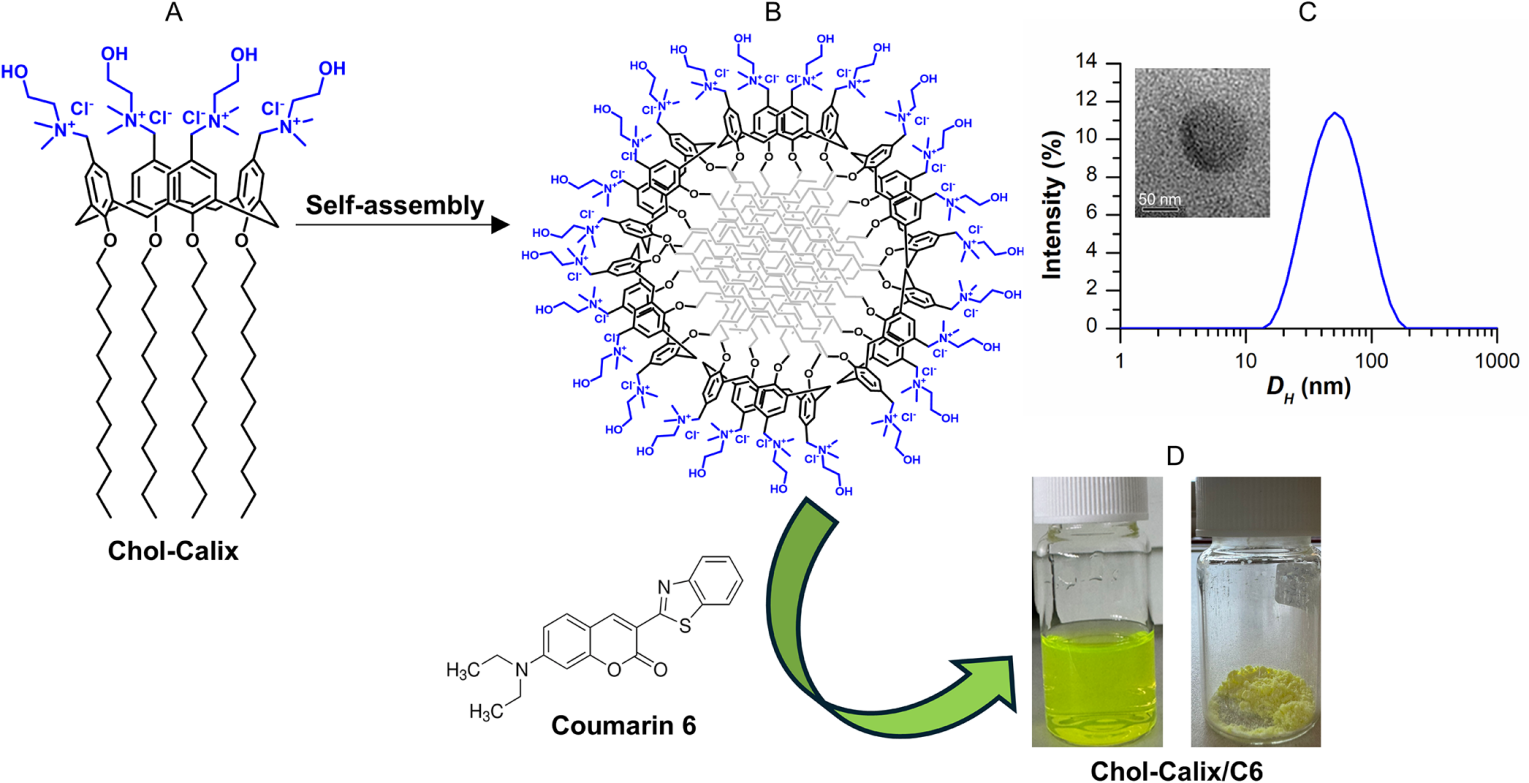
(A) Chemical structure of Chol-Calix. (B) Schematic representation of the Chol-Calix nanostructure. (C) Dynamic light scattering spectrum (intensity-weighted distribution) and TEM image (inset) of the Chol-Calix nanoaggregates. Entrapment of coumarin 6 and (D) photographs of Chol-Calix/C6 as a colloidal dispersion (left) and freeze-dried powder (right bottom).

Owing to its amphiphilic structure, Chol-Calix self-assembles into nanostructures (Figure 1B) in a biomimetic medium such as phosphate-buffered saline (10 mM, pH 7.4) at concentrations above approximately 8 μM.^27^ Dynamic Light Scattering (DLS) measurements revealed the formation of aggregates with a mean hydrodynamic diameter of ∼50 nm (Figure 1C), a polydispersity index of 0.2, and a zeta potential (ζ) of +24.7 mV. Transmission Electron Microscopy (TEM) images corroborated the size of the nanoaggregates and showed a micelle-like morphology with a quasi-spherical shape (Figure 1C, inset).

The choline moieties were specifically designed to confer the nanostructure the ability to recognize and bind choline transporters, which are overexpressed on the surface of various tumor cells.

Entrapment of C6 (Figure 1) in the nanocarrier was achieved using a straightforward phase solubility method (see Experimental Section), and the enhancement of C6 water solubility was immediately apparent from the yellow color of the resulting colloidal dispersion (Figure 1D). In contrast, when C6 alone underwent the same treatment, a colorless sample was obtained, consistent with the very low solubility of C6 in aqueous media. This behavior was confirmed by the absorption spectra of the Chol-Calix/C6 nanosystem and C6 alone (Figure 2A).

**Figure 2:**
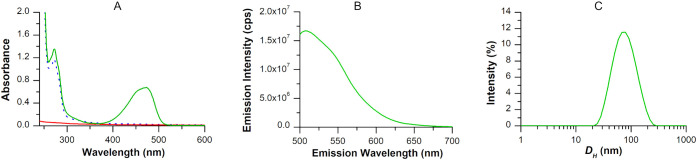
Characterization of Chol-Calix/C6 water dispersion (0.6 mM Chol-Calix and 18.7 μM C6): (A) UV–vis spectrum (green line) in comparison with Chol-Calix (dotted blue line) and C6 alone (red line). (B) Fluorescence spectrum (Exc 465 nm, Em 507 nm). (C) Dynamic light scattering spectrum (intensity-weighted distribution mode).

The absorption spectrum of Chol-Calix/C6 showed the characteristic bands of Chol-Calix at 210 and 270 nm and of C6 at 470 nm (Figure 2A), while the fluorescence spectrum confirmed the emission band of C6 at 501 nm (Figure 2B). The quantum yield of Chol-Calix/C6 in water was 0.27, lower than that of C6 in ethanol (0.66), likely due to interactions between C6 moieties packed in close proximity to the calixarene cavities, as supported by molecular modeling simulations (Figure 3).

**Figure 3:**
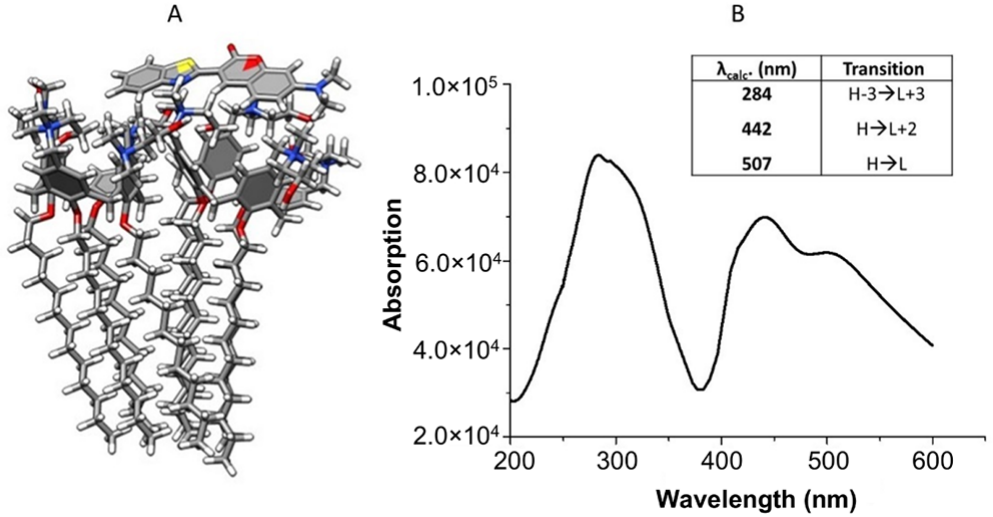
Molecular modeling: (A) Chol-Calix/C6_1 geometry and (B) UV–vis simulated optical absorption spectrum for Chol-Calix/C6_1 at the CAM-B3LYP/6-31G­(d)/SMD-water level (insets: absorption and main contribution to the transition of simulated models).

The drug loading capacity (%) was calculated to be approximately 0.7%, corresponding to a concentration of C6 entrapped in the nanocarrier of 6.5 μg/mL. This concentration is sufficient for biological applications, as C6 levels in the range of 0.25–5 μg/mL have been reported to be effective for cell imaging.

Dynamic Light Scattering (DLS) measurements (Figure 2C) revealed that Chol-Calix/C6 forms nanoaggregates with a mean hydrodynamic diameter of approximately 77 nm (Z average) and a polydispersity index of 0.3 indicative of a sample with a moderate to broad nanoparticle size distribution. The nanoaggregates exhibited a positively charged surface, as indicated by a zeta potential (ζ) of +22.5 mV, which is sufficiently high to promote colloidal stability by preventing aggregation through electrostatic repulsion.

### 3.2 Molecular Modeling Simulations

Computer modeling simulations were carried out to gain insights into the interactions between the dye and the nanocontainer. The modeling simulations were performed as described in the Supporting Information starting from four different geometries composed by two Chol-Calix units and one C6 molecule interacting with them. The free energy values (ΔGf) associated with the formation of the Chol-Calix/C6 nanostructured models in an aqueous solution were calculated. The data clearly indicated a more efficient binding for the bridged models Chol-Calix/C6_1 (ΔGf = −22.81 kcal·mol^–1^) and Chol-Calix/C6_2 (ΔGf = −21.38 kcal·mol^–1^) compared to the Chol-Calix/C6_3 (ΔGf = −11.29 kcal·mol^–1^) and Chol-Calix/C6_4 (ΔGf = −16.84 kcal·mol^–1^) structures (Figure S3). Optimization unequivocally demonstrates that the Chol-Calix/C6 complex reorganizes into its most stable form, in which the C6 moiety acts as a bridge between calixarene cavities (Figure 3A). The Chol-Calix/C6_3 and Chol-Calix/C6_4 geometries, where the C6 unit is sandwiched between the lipophilic chains and within the cavities of calixarene molecules, represent the less energetically favorable configurations.

The simulated absorption spectrum for Chol-Calix/C6_1 depicted in Figure 3B, confirms the energy data reported in the table of Figure 3B. In detail, the Chol-Calix/C6_1 geometry exhibits an intense absorption band in the region from 400 to 450 nm according to the experimental optical absorption spectrum (Figure 2A). In addition, an absorption in the region 250–350 nm, with a maximum at 282 nm, was calculated according to the experimental spectra. In contrast, the Chol-Calix/C6_3 and Chol-Calix/C6_4 geometries showed a single absorption band around 400 nm (transition H → L). Regarding the Chol-Calix/C6_2 structure, the UV data evolve into intense absorption bands in the region from 400 to 450 nm and in the region 250–350 nm similar to the Chol-Calix/C6_1, according to the same energy data reported in the table of Figure 3B.

### 3.3 Stability of Chol-Calix/C6

The stability of the fluorescent nanosystem, stored both at room temperature and at 4 °C, was monitored over time (Table 1).

**Table 1:** Monitoring of Chol-Calix/C6 Stability by Evaluation of Absorbance, Emission Intensity, and Size over Time, at 25 and 4 °C

		*t*_3 months_		*t*_6 months_	
	*t*_0_	25 °C	4 °C	25 °C	4 °C
Absorbance λ_470_ nm	0.72	0.72	0.72	0.69	0.71
Emission λ_507_ nm	17 × 10^6^	16 × 10^6^	16 × 10^6^	15.5 × 10^6^	16 × 10^6^
Z average (nm)	77.42	99.81	97.28	90.10	94.64
PDI	0.31	0.45	0.40	0.40	0.45

After six months from preparation, measurements of absorbance, fluorescence emission, and size evidenced a good stability. Notably, the nanosystem exhibited superior stability compared to C6 loaded in polymeric nanoparticles, whose quantum yield stability was reported to last over than two months in powder form and only up to 3 days in aqueous medium.^13^

The nanosystem also demonstrated high stability upon freeze-drying without the use of cryoprotectants. The lyophilized powder (Figure 1D), upon resuspension in water, yielded a yellow colloidal dispersion that retained the initial (*t*_0_) values for all parameters reported in Table 1.

### 3.4 Photodynamic Properties of Chol-Calix/C6

Coumarin derivatives are attractive photosensitizers due to their intriguing photophysical properties, including strong fluorescence, two-photon absorption, facile intersystem crossing, long triplet-state lifetime, and stimuli responsiveness.^17^ These features enable effective energy/charge transfer processes, making coumarins suitable for redox-responsive photodynamic therapy, which is one of the most promising localized and noninvasive treatments for cancer^51^ and infections.^52^

To explore the potential photosensitizing properties of Chol-Calix/C6, we evaluated its ability to photodegrade methylene blue (MB) upon irradiation with laser light at 470 nm under both aerated (presence of O_2_) and deaerated (absence of O_2_) conditions.

Experimental evidence suggests that the Chol-Calix/C6 nanosystem possesses photosensitizing properties upon light excitation. A 470 nm light source was properly selected because Chol-Calix/C6 exhibits significant absorption at this wavelength, whereas MB shows negligible absorption. Figure 4A and B illustrates the optical spectral changes of the Chol-Calix/C6/MB aqueous dispersion (Abs_470 nm_= 0.7) upon photoexcitation with a source laser at 470 nm (800 mW) at different irradiation times (0, 2, 4, 6, 8, 10, 20, 30, 40, and 60 min) under aerated and deareated conditions, respectively. The decrease of the absorption band of MB at around 665 nm confirms its photodegradation induced by the excited state of Chol-Calix/C6, while the decrease of the absorption band at around 469 nm indicates photodegradation of the Chol-Calix/C6 nanosystem. Upon photoexcitation of Chol-Calix/C6, the neoformed excited state [Chol-Calix/C6]* induces direct photodegradation of MB. Under deaerated conditions, ∼90% of MB was degraded after 15 min (Figure 4C, red line). Conversely in the presence of O_2_, MB photodegradation was slower according to the quenching of the excited state [Chol-Calix/C6]* by the O_2_ species, reaching ∼60% after 30 min (Figure 4C, black line).

**Figure 4:**
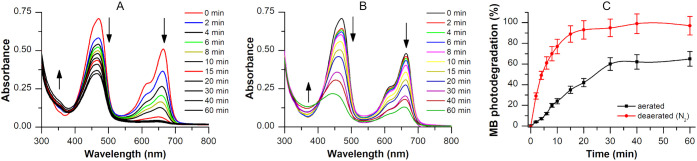
Photoinduced degradation of methylene blue (MB) (2 μL, Abs 0.6) in the dispersion of Chol-Calix/C6 (Abs 0.7) upon irradiation with a source laser at 470 nm and power 800 mW, at different irradiation time: (A) optical absorption spectral changes of the Chol-Calix/C6/MB dispersion upon photoexcitation in deaerated condition; (B) optical absorption spectral changes of the Chol-Calix/C6/MB dispersion upon photoexcitation in aerated condition; (C) percentage of MB photodegradation under aerated (black line) and deaerated (red line) conditions.

### 3.5 Biological Assays

#### 3.5.1 Western Blot Analyses

As a charged molecule, choline cannot freely cross the lipid bilayer of cell membranes, and its uptake relies on specific protein transporters (CHT1, OCT1, OCT2, CTL1–5). In cancer, choline metabolism becomes dysregulated not only due to the overexpression of metabolic enzymes but also as a result of altered signaling pathways that enhance choline uptake and utilization.^34^ This leads to an increased expression of choline transporters in cancer cells compared to noncancerous cells.^53^

Based on this, we investigated the Chol-Calix/C6 nanosystem as a fluorescent agent for imaging human hepatocellular carcinoma (HCC) and breast carcinoma (BCa) cells. HCC is the sixth leading cause of cancer-related death worldwide, while BCa is the second leading cause of cancer-related death in women, both showing increasing incidence.^54,55^ Despite advances in therapeutic strategies, HCC and BCa remain associated with poor prognoses due to late-stage diagnosis, high recurrence rates, and elevated mortality. Therefore, the development of novel diagnostic approaches is critically needed.

To detect differences in the expression level of the choline transporter-like protein 1 (CTL1), also known as SLC44A1 and CD92, Western blot analysis was performed in cancer and nonmalignant cells. In the BCa model, MCF-7 cells showed a higher expression of choline receptor protein than MDA-MB-231 cells. Notably, normal human dermal fibroblasts (HuDe) did not express the receptor (Figure 5). In the HCC model, the Hep3B cells exhibited the highest expression level of choline receptor protein among all HCC cells analyzed, whereas the SNU398 cells showed the lowest expression (Figure 5).

**Figure 5:**
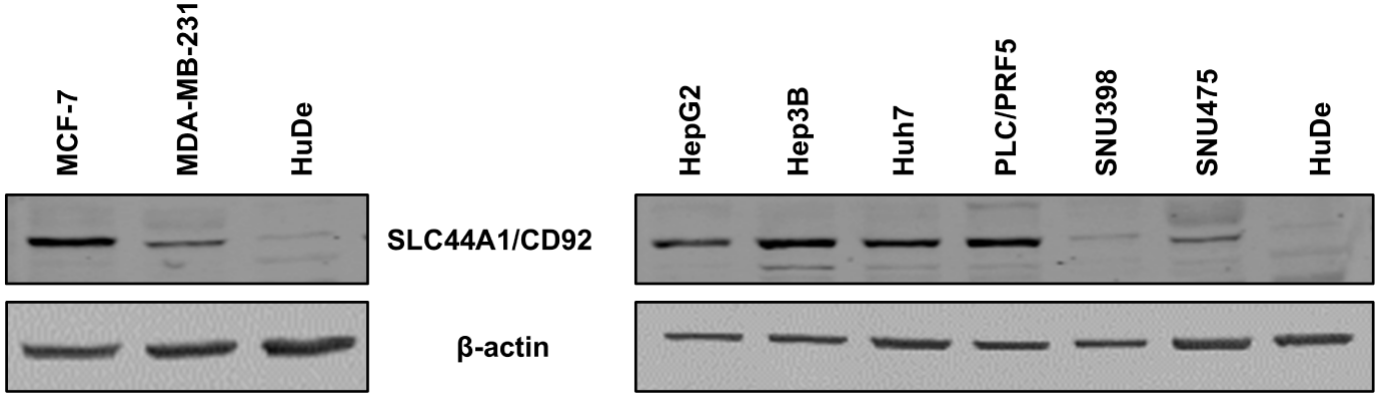
Western blotting analysis for choline transporter expression on breast carcinoma cells (MCF-7 and MDA-MB-231), hepatocellular carcinoma cells (HepG2, Hep3B, Huh7, PLC7PRF5, SNU398, and SNU475), and normal human dermal fibroblast (HuDe) cells.

#### 3.5.2 Cellular Uptake

Fluorescence microscopy revealed differential cellular uptake of the Chol-Calix/C6 nanosystem among different cell lines, which was in accordance with the cellular choline-like transporter expression levels (Figures 6 and 7). Following incubation with Chol-Calix/C6, Hep3B and MCF-7 cells displayed strong fluorescence signals, which appeared distributed diffusely and as cytoplasmic dot-like structures (Figures 6 and 7).

**Figure 6:**
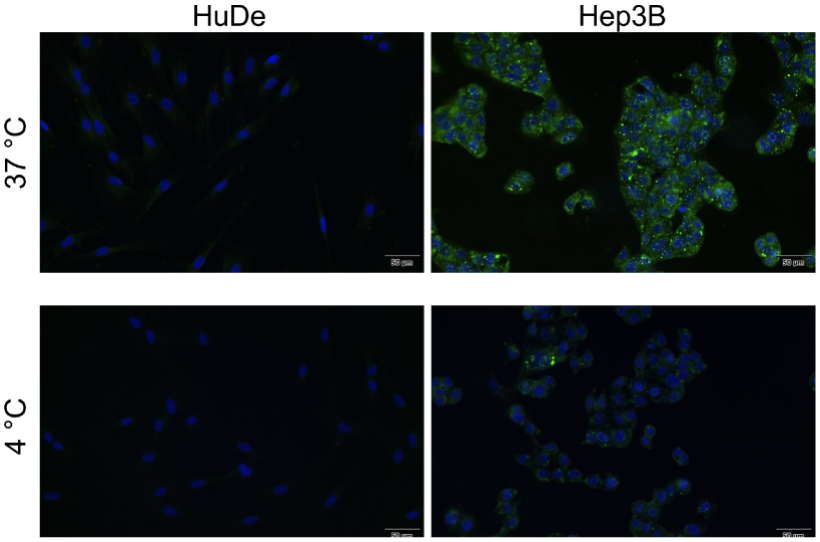
Fluorescence images of HuDe and HCC cells treated with Chol-Calix/C6 (25 μM Chol-Calix, 0.77 μM C6) for 1 h at 37 and 4 °C. In green is the coumarin C6 fluorophore. In blue, nuclei stained with DAPI. Scale bar = 50 μm.

**Figure 7:**
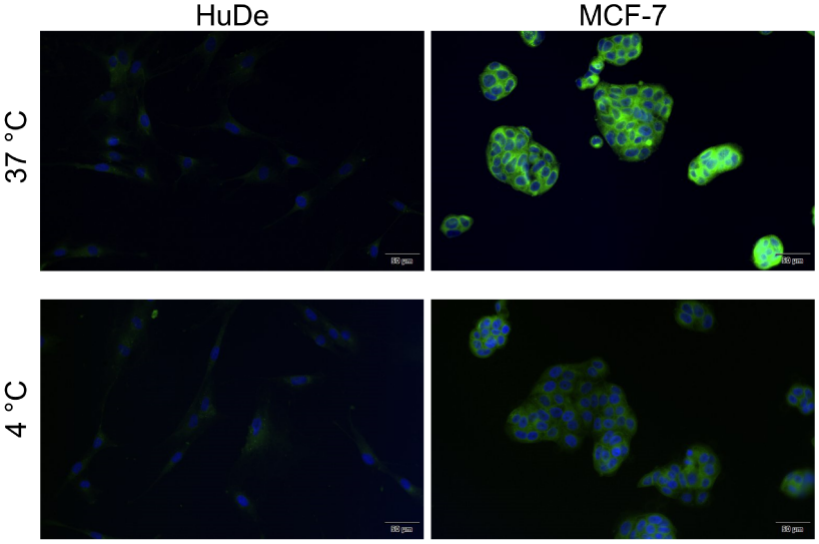
Fluorescence images of HuDe and BCa cells treated with Chol-Calix/C6 (25 μM Chol-Calix, 0.77 μM C6) for 1 h at 37 and 4 °C. In green, the C6 fluorophore. In blue, nuclei stained with DAPI. Scale bar = 50 μm.

Additionally, a distinct plasma-membrane-associated fluorescence pattern was observed. In contrast, SNU398 and MDA-MB-231 cells, which have lower transporter expression, showed markedly weaker fluorescence, primarily localized as intracellular dots (Figure S4). Importantly, no fluorescence signal was detected in nonmalignant HuDe cells (Figures 6 and 7).

These findings indicate that the binding and uptake of the Chol-Calix/C6 nanosystem correlate with choline transporter expression levels and that fluorescence intensity effectively distinguishes cancerous cells from normal cells.

To confirm the involvement of a transporter-mediated cellular uptake, the assays were also performed at 4 °C. It is well established that transporter-mediated endocytosis is an active process occurring optimally at 37 °C, and that lower temperatures impair transporter activity and ligand binding.^56^ Incubation at 4 °C led to a significant reduction in fluorescence intensity in both cancer cell types (Figures 6 and 7), supporting the hypothesis that Chol-Calix/C6 uptake occurs, at least in part, through an active transporter-mediated mechanism.

To further support the involvement of the choline transporter in the cellular uptake of Chol-Calix/C6, competition experiments were performed in MCF-7 cells. MCF-7 cells were pretreated with choline at varying concentrations before the treatment with Chol-Calix/C6 (Figure 8).

**Figure 8:**
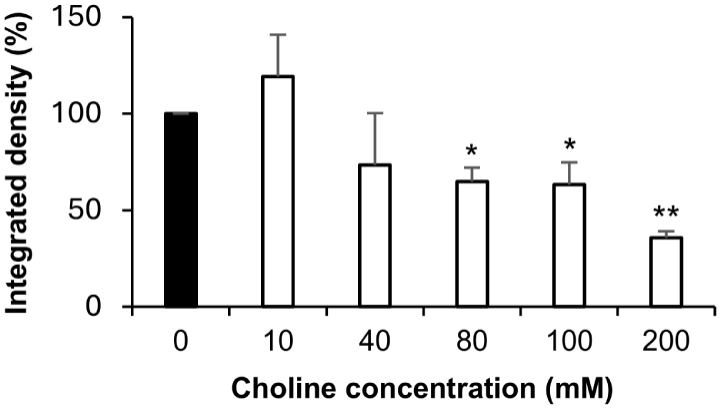
Free choline competes with Chol-Calix/C6 for cellular uptake in the MCF-7 cells. Cells were incubated for 30 min with different amounts of choline (40, 80, 100, and 200 mM), then treated with Chol-Calix/C6 (25 μM Chol-Calix, 0.77 μM C6) for 1 h. Data shown are means ± S.E.M., representative of three independent experiments, and expressed as percentage of fluorescence in MCF-7 cells incubated with Chol-Calix/C6 alone. *p < 0.05, **p < 0.0005 compared to control MCF-7 cells not incubated with free choline (*t* student analysis).

A reduction in fluorescence intensity in MCF-7 cells treated with Chol-Calix/C6 following choline pretreatment indicated that choline competes for binding to the transporter. A significant decrease in Chol-Calix/C6 uptake was observed already at 80 mM and increased with higher choline concentrations, resulting in a fluorescence reduction of 65% at a 200 mM choline concentration. This suggests that the nanosystem binds more effectively to choline transporters than free choline units, likely due to multiple interactions. The multivalency effect is commonly employed in nature to enhance specificity, selectivity, and avidity in molecular recognition processes.^57,58^ Overall, these findings support a choline-transporter-mediated mechanism for the cellular uptake of Chol-Calix/C6, highlighting its potential for imaging tumor cells that overexpress choline transporters.

#### 3.5.3 Chol-Calix/C6 Photodynamic Activity on Cancer Cells

PDT is an FDA-approved modality of cancer therapy involving the selective photosensitization of neoplastic cells by photosensitizers, with a highly localized eradication of neoplastic lesions and minimal damage to adjacent tissues.^59^

To the best of our knowledge, most known uses of C6 are limited to fluorescence tracing. C6 is not typically recognized as a photosensitizer and only C6 metal complexes^60^ have been reported in the literature as photosensitizers.

To evaluate the potential of Chol-Calix/C6 in PDT application, MCF-7 cells previously incubated with Chol-Calix/C6 were subjected to irradiation (10 min) with a laser light (λ 470 nm).

Cell viability assay demonstrated that in MCF-7 cells treated with the nanosystem, but not irradiated, cell viability was not affected, highlighting the noncytotoxicity of Chol-Calix/C6 (Figure 9). Only a 14% inhibition of cell viability was observed in the untreated cell after irradiation, whereas a complete loss of cell viability was instead detected in the treated cells upon irradiation.

**Figure 9:**
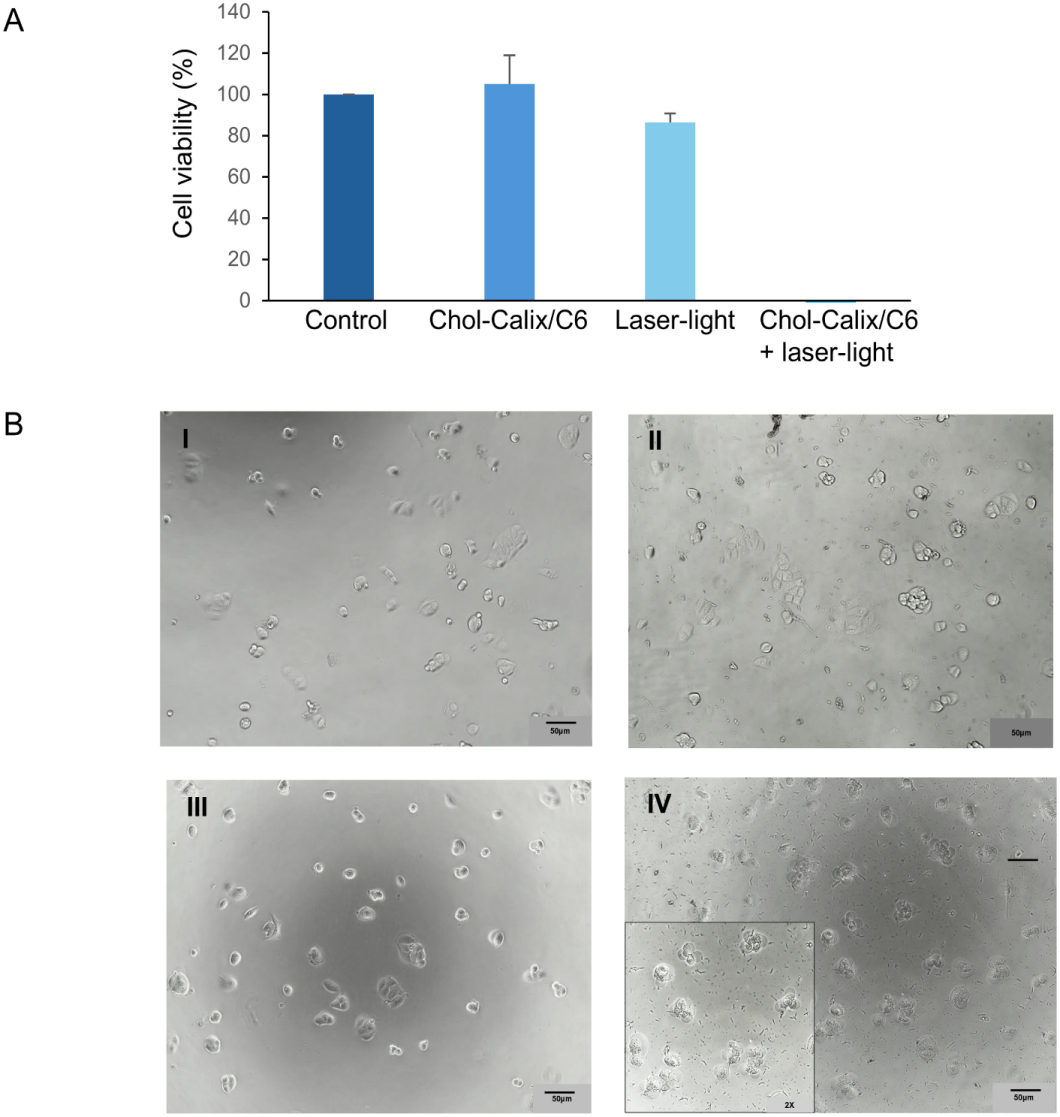
Photodynamic Activity of Chol-Calix/C6. (A) Cell viability of MCF-7 cells nontreated (control) and treated with Chol-Calix/C6 (25 μM Chol-Calix, 0.77 μM C6) for 1 h at 37 °C, followed by irradiation with laser light (λ = 470 nm), was assessed by MTS assay after 24 h. Data are expressed as the percentage of control cells and are the means ± SD of three separate experiments. (B) Bright-field images of MCF-7 cells: untreated (I), treated with Chol-Calix/C6 (II), irradiated only (III), and treated with Chol-Calix/C6 followed by irradiation (IV). Inset: 2-fold magnification of a selected area from panel IV.

Temperature monitoring by a thermocamera excluded a photothermic effect; indeed, the temperature was maintained in the range of 32–35 °C. These data strongly supported the Chol-Calix/C6 nanosystem as a potential novel nanotheranostic agent, capable to visualize cancer cells overexpressing choline transporters and damage them with a punctual light-triggered control.

## 4 Conclusions

A novel fluorescent nanosystem was successfully developed by entrapping the hydrophobic dye Coumarin 6 in calix[4]­arene-based nanostructures, exposing choline ligands (Chol-Calix). Comprehensive physicochemical and photophysical characterization evidenced that the nanosystem possesses nanoscale size, good fluorescence quantum yield, remarkable stability in both aqueous dispersion and lyophilized form, and phototriggered photodynamic activity. Biological assays demonstrated that the nanosystem exhibits selective internalization in cancer cells overexpressing choline transporters (MCF-7 and Hep3B), in contrast to nonmalignant fibroblasts (HuDe) that showed negligible uptake. Intracellular fluorescence intensity correlated with choline transporter expression levels, and competitive inhibition assays supported a transporter-mediated cancer cell uptake mechanism. Importantly, beyond selective cancer cell imaging capability, the nanosystem displayed an effective photodynamic activity, inducing cancer cell death exclusively upon visible-light irradiation. Overall, the fluorescent nanosystem appears to be a promising photoresponsive nanotheranostic platform with potential applications in precision tumor cell imaging and site-specific light-controlled treatment of cancer, including imaging-guided surgery and ex vivo cancer cell detection.

## Supplementary Material



## Data Availability

Data will be made available on request.

## Notes

Experimental details for the multistep synthesis of Chol-Calix and the synthetic scheme (Figure S1); ^1^H NMR spectrum of Chol-Calix (Figure S2); additional details on molecular modeling simulations of Chol-Calix/C6 interactions and corresponding images (Figure S3); fluorescence images of HCC (SNU398) and BCa (MDA-MB-231) cells treated with Chol-Calix/C6 (Figure S4) (PDF)
