# Supplementary material for: Calixarene-Based Nanostructures for Delivering Coumarin 6 for Tumor-Cell Imaging and Photoinduced Toxicity
Source: ACS Appl Nano Mater. 2026 Feb 6;9(10):4493–503. doi: 10.1021/acsanm.5c05263 (PMC12993873; doi:10.1021/acsanm.5c05263)
Supplement: Supplementary file 1 [file an5c05263_si_001.pdf]

Supporting Information

Calixarene-Based Nanostructures for Delivering Coumarin 6 for Tumor-Cell Imaging and Photo-Induced Toxicity

Loredana Ferreri,[†] Giuseppe Granata,[†] Giuseppe Forte,[‡] Melchiorre Cervello,[$] Antonella Cusimano,[$*] Salvatore Petralia,[‡†*] and Grazia Maria Letizia Consoli[†*]

[†]*Institute of Biomolecular Chemistry - C.N.R., Catania 95126, Italy*

[‡]*Department of Drug and Health Sciences, University of Catania, Catania 95125, Italy*

[$]*Institute for Research and Biomedical Innovation - C.N.R., Palermo 90146, Italy*

Email:

antonella.cusimano@cnr.it

salvatore.petralia@unict.it

graziamarialetizia.consoli@cnr.it

CONTENT

Experimental details for the multistep synthesis of CholCalix.

Figure S1. Synthetic scheme for the preparation of CholCalix.

Figure S2. ^{1}H-NMR spectrum of CholCalix.

Molecular modelling simulations of Chol-Calix/ C6 interactions.

Figure S3. Simulation modelling for CholCalix/C6 interaction systems.

Figure S4. Fluorescence images of HCC (SNU398) and BCa (MDA-MB-231) cells treated with Chol-Calix/C6.

Experimental details for the multistep synthesis of CholCalix

CholCalix was synthesized by a multistep procedure starting from the commercial *p*-H-calix[4]arene derivative (**Figure S1**) and reported in literature. All the intermediate compounds were characterized by ^{1}H NMR spectra that showed signals consistent with the expected structures and assignments reported in literature. [1,2,3]

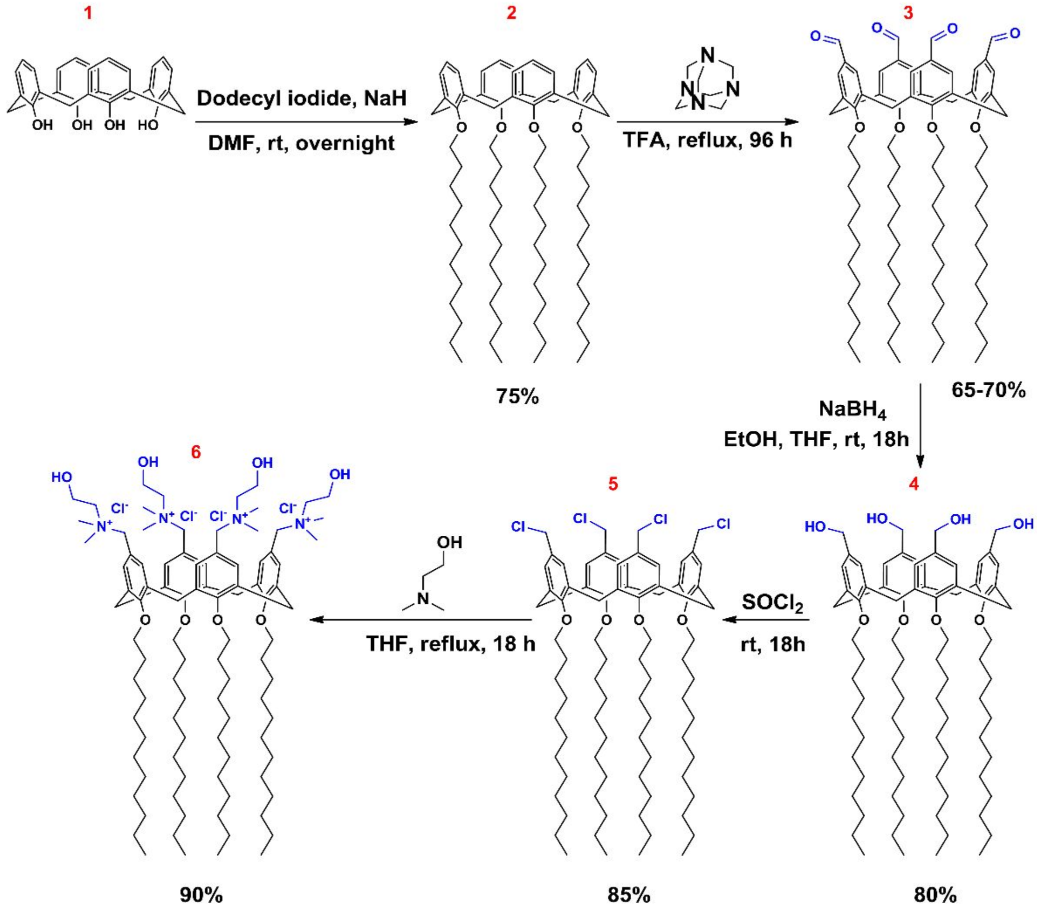

Figure S1. Procedure for the synthesis of Chol-Calix.

25,26,27,28-Tetradodecyloxycalix[4]arene (2)

p-H-Calix[4]arene **(1)** (5.72 g, 13.5 mmol) and 5.4 g NaH (60% dispersion in oil, 135 mmol) was stirred 140 mL of dry DMF for 15 min under argon. Dodecyl iodide (33.2 mL, 135 mmol) was then added and the mixture was stirred at 75 °C overnight. DMF was removed under vacuum, 0.5 N HCl (200 mL) was added. After stirring overnight, the solid was recovered by filtration and washed several times with MeOH and small portion of acetone. This operation can be repeated to reach the desired purity of compound **2**.

5,11,17,23-tetra-formyl-25,26,27,28-tetra-dodecyloxy-calix[4]arene (3)

Under an inert gas atmosphere a mixture of compound **2** (3.3 g, 3 mmol) and tetramine (16.2 g, 120 mmol) in CF_3COOH (100 mL) was stirred for 96 h under reflux. The mixture was cooled to room

temperature and then poured into a stirring solution of 2 M HCl (200 mL) and CH_2Cl_2 (200 mL), and vigorously stirred for 1 h. The mixture was extracted with CH_2Cl_2 (2 x 100 mL) and the combined organic layers were washed with saturated aqueous Na_2CO_3 (2 x 100 mL) and brine (2 x 100 mL), dried over sodium sulfate and the solvent was then removed under reduced pressure. The raw product was further purified by column chromatography (silica gel 60; hexane: ethyl acetate: CH_2Cl_2, 7:3:2) to give compound **3** as a white solid.

5,11,17,23-tetra-hydroxymethyl-25,26,27,28-tetra-dodecyloxy-calix[4]arene (4) Under an inert gas atmosphere ethanol (50 mL) was added to a stirring solution of **3** (2.73 g, 2 mmol) in THF (10 mL). $NaBH_4$ (2.6 g, 70 mmol) was then added and the mixture stirred for 18 h at room temperature. The mixture was then concentrated under vacuum and the resulting solid dissolved in CH_2Cl_2 (100 mL). 2 M HCl (100 mL) was added slowly and the solution was stirred for 1 h. The reaction mixture was then extracted with CH_2Cl_2. The combined organic fractions were washed with 2 M HCl (3 x 100 mL) and dried over Na_2SO_4. The solvent was removed *in vacuo* to give compound **3** as a white solid.

5,11,17,23-tetra-methylchloride-25,26,27,28-tetra-dodecyloxy-calix[4]arene (5) Under an inert gas atmosphere thionyl chloride (8 mL) was added to calix[4]arene derivative **4** (2 g, 1.6 mmol) and then stirred at room temperature for 18 h. The mixture was concentrated under vacuum and the resulting solid was dissolved in CH_2Cl_2 (150 mL), washed with saturated aqueous Na_2CO_3 (3 x 150 mL) and dried over Na_2SO_4. The solvent was removed under reduced pressure to give compound **5** as a white solid.

5,11,17,23-Tetra(*N,N*-dimethyl-*N*-hydroxyethylammonium)-methylene-25,26,27,28-tetradodecyloxycalix[4]arene tetrachloride (6)
A solution of *N,N*-dimethylethanolamine (1.3 g, 1.5 mL, 14.9 mmol) in THF (15 mL) was added to a stirring solution of **5** (4.1 g, 3.2 mmol) in THF (60 mL). The reaction mixture was refluxed for 24 h. After cooling, the suspension was centrifuged at 4000 rpm, 5 min. The precipitate was washed with THF (40 mL) and with acetonitrile (4 x 20 mL) by repeated centrifugation (4000 rpm, 5 min) and removal of the solvent. The solid precipitate was recovered and dried under vacuum to give compound **6** as a white solid.

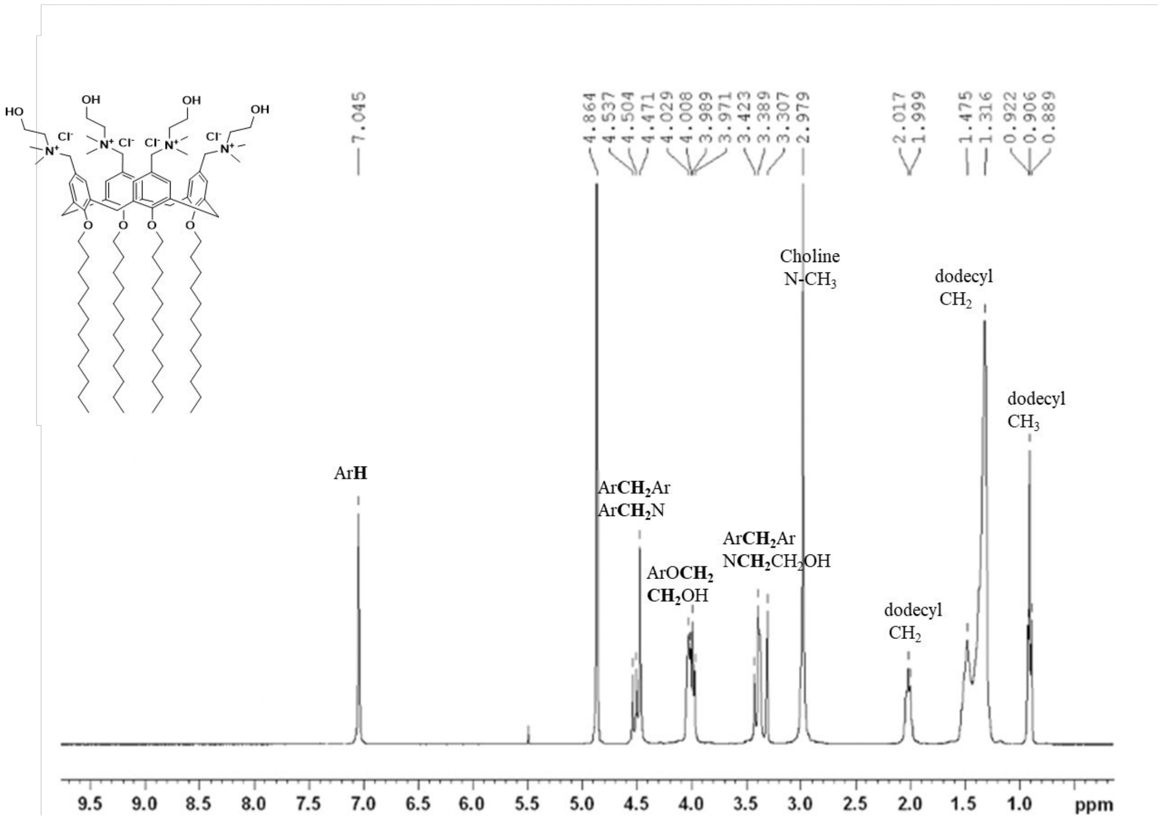

Figure S2. ^{1}H-NMR spectrum of Chol-Calix (400.13 MHz, MeOD, 297 K).

Molecular modelling simulations of Chol-Calix/ C6 interactions.

The starting geometry is composed by two CholCalix units and one single interacting coumarin molecule were examined. Four different geometries habe been selected as starting gemoetries: in CholCalix/C6_1, the coumarin molecule is positioned horizontally between the two calixarene cups, acting a s a bridge. In CholCalix/C6_2, the coumarin is inserted vertically into the cup of a single calixarene molecule. In CholCalix/C6_3, the coumarin is placed among the lipophilic chains of two calixarene molecules. Finally, in CholCalix/C6_4, the coumarin lies at the base of the lipophilic chains of the calixarene, mimicking an intramicellar environment (**Figure S3**). Calculations were performed at at CAM-B3LYP/6-31G(d)/PCM-water level.

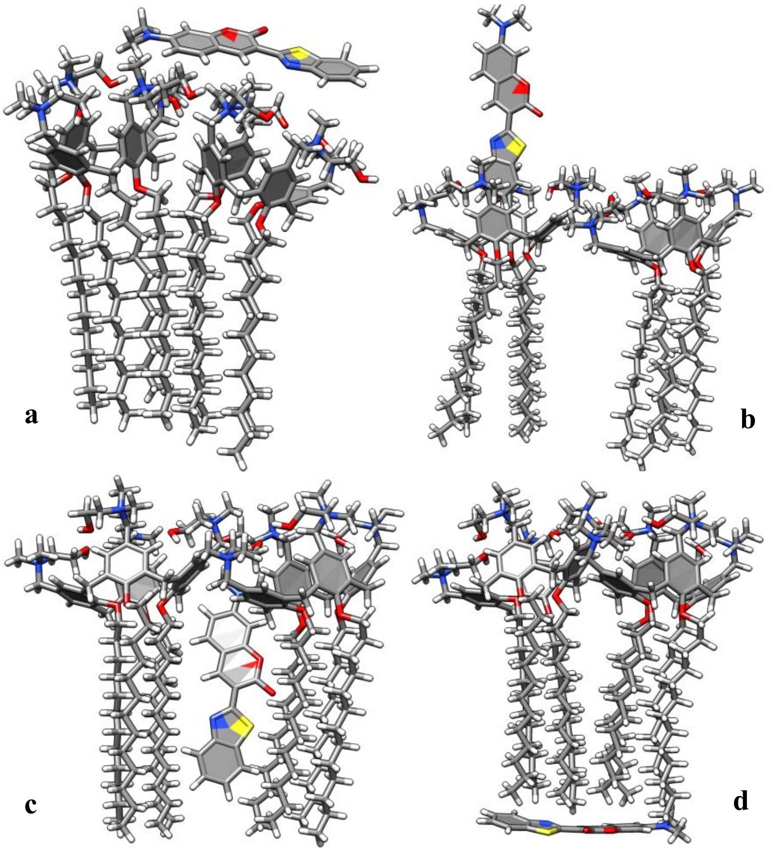

Figure S3. Simulation modelling for CholCalix/C6 interaction systems: bridge CholCalix/C6_1 model (a); CholCalix/C6_2 coumarin inserted into the cup of a single calixarene molecule model (b); CholCalix/C6_3 sandwich model (c), and CholCalix/C6_4 coumarin placed at the base of the lipophilic chains of the calixarene (d). In figures only one CholCalix unit is reported for clarity.

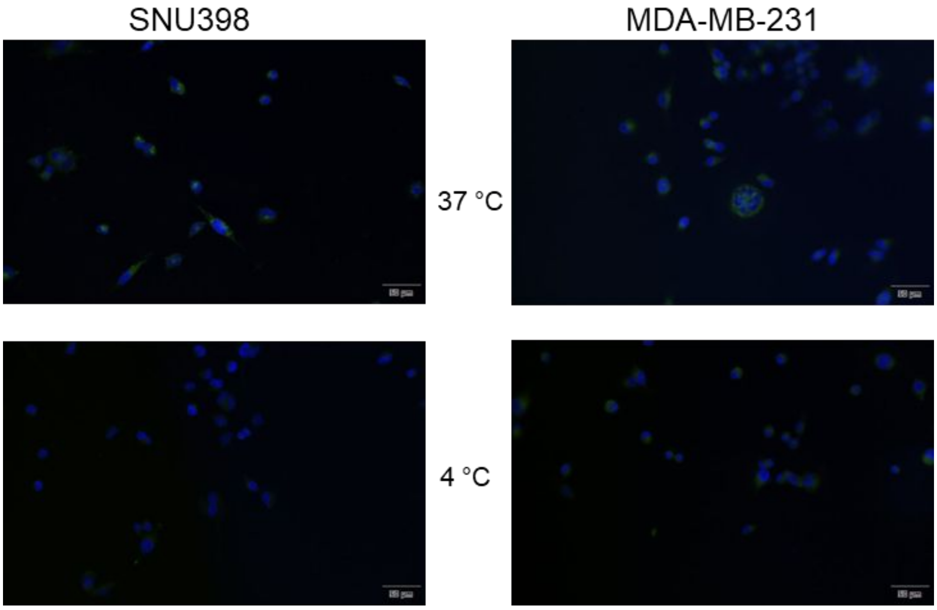

Figure S4. Fluorescence images of HCC (SNU398) and BCa (MDA-MB-231) cells treated with Chol-Calix/C6 (25 μM Chol-Calix, 0.77 μM) for 1h at 37°C and 4°C).

References

[1] Rodik, R. V.; Anthony, A. S.; Kalchenko, V. I.; Mélya, Klymchenko, Y. A. *New J.* Chem. **2015**, *39*, 1654.

[2] Consoli, G. M .L.; Di Bari, I.; Blanco, A. R.; Nostro, A.; D'Arrigo, M.; Pistarà, V.; Sortino, S. *ACS Med. Chem. Lett.* **2017**, *8*, 881.

[3] Granata, G.; Paterniti, I.; Geraci, C.; Cunsolo, F.; Esposito, E.; Cordaro, M.; Blanco, A. R.; Cuzzocrea, S.; Consoli, G. M. L. *Mol. Pharm.* **2017**, *14*, 1610.
